# Nasopharyngeal carriage rate, serotype distribution, and antimicrobial profiles of Streptococcus pneumoniae among patients with acute respiratory tract infection in Manado, North Sulawesi, Indonesia

**DOI:** 10.1099/acmi.0.000703.v4

**Published:** 2024-03-15

**Authors:** Diana Shintawati Purwanto, Miftahuddin Majid Khoeri, Wisnu Tafroji, Stefana Helena Margaretha Kaligis, Rocky Wilar, Billy Johnson Kepel, Hessyani Patricia Theodora Raranta, Lidia Gaghiwu, Sven Hammerschmidt, Waode Fifin Ervina, Dodi Safari

**Affiliations:** 1 Department of Biochemistry, Faculty of Medicine, Sam Ratulangi University, Manado, North Sulawesi, Indonesia; 2 Department of Clinical Laboratory, R. D. Kandou General Hospital, Manado, North Sulawesi, Indonesia; 3 Eijkman Research Center for Molecular Biology, National Research and Innovation Agency, Cibinong, West Java, Indonesia; 4 Department of Pediatrics, Faculty of Medicine, Sam Ratulangi University / R. D. Kandou General Hospital, Manado, North Sulawesi, Indonesia; 5 Department of Chemistry, Faculty of Medicine, Sam Ratulangi University, Manado, North Sulawesi, Indonesia; 6 Department of Molecular Genetics and Infection Biology, Interfaculty Institute for Genetics and Functional Genomics, Center for Functional Genomics of Microbes, University of Greifswald, Greifswald, Germany

**Keywords:** acute respiratory infection, Indonesia, Streptococcus pneumoniae

## Abstract

We studied the carriage rate, distribution of serotype, and antimicrobial profile of *Streptococcus pneumoniae* (*S. pneumoniae*) among patients with acute respiratory tract infections (ARTI) in two primary health centres and a tertiary referral hospital from 2019 to 2020 in Manado, North Sulawesi, Indonesia before 13-valent pneumococcal conjugate vaccine (PCV13) introduction. A total of 106 nasopharyngeal swab samples were collected from children and adult patients. Serotyping of *S. pneumoniae* strain was performed by sequential multiplex PCR and Quellung reaction. Antimicrobial profile was performed by the disc diffusion method. We identified thirty-one patients carried *S. pneumoniae strains* (29 %). The *S. pneumoniae* carriage rate was found to be higher among children aged 2–5 years (13/32; 40.6 %) than in children under 1 year (8/27; 29.6 %), children and adolescents under 18 years of age (5/20; 25.0 %) and adult patients (5/27; 18.5 %). The distribution of serotypes varied, including 14, 18C, 19A, 23F, 19F and 35B (two strains each) and 1, 3, 6B, 6C, 31, 9V, 15C, 16F, 17F, 23A, 35F (one strain each) and non-typeable (9/31; 29 %). We found *S. pneumoniae* isolates were susceptible to vancomycin (30/31; 97 %), chloramphenicol (29/31; 94 %), clindamycin (29/31; 94 %), erythromycin (22/31; 71 %), azithromycin (22/31; 71 %), tetracycline (14/31; 45 %), penicillin (11/31; 35 %), and sulfamethoxazole/trimethoprim (10/31; 32 %). This study provides supporting baseline data on distribution of serotype and antimicrobial profile of *S. pneumoniae* among patients with ARTI before PCV13 introduction in Manado, North Sulawesi, Indonesia.

## Data Summary

All data on distribution of serotype and antimicrobial profiles of *S. pneumoniae* strains are available in Table S1, available in the online version of this article.

## Introduction

*Streptococcus pneumoniae* (*S. pneumoniae*) or pneumococcus is a Gram-stain-positive bacterium able to asymptomatically colonize the nasopharynx of humans. The nasopharynx is the primary reservoir for the pneumococcus and a major source of transmission for the bacterium to spread into the community [1]. Prolonged pneumococcal nasopharyngeal carriage can in due course progress into invasive pneumococcal disease [1]. This opportunistic pathogen can cause severe disease when sterile sites such as the lungs, bloodstream, and meninges are encountered [2]. Viral upper respiratory tract infections often predispose to bacterial pneumonia as well, most likely by facilitating the invasion of bacteria such as the pneumococci colonizing the nasopharynx [3]. Pneumococcal disease has higher prevalence in individuals who have a weakened immune system such as young children, elderly adults, and the immunocompromised [4].

Several epidemiological data on *S. pneumoniae* carriage have been reported from the population in various regions of Indonesia [5,10]. Safari, D, *et al*. reported that *S. pneumoniae* strains were isolated from 37 of 159 adult and children outpatients with ARTI (18.5 %) at a district hospital in Tabanan, Bali, Indonesia, in 2017 with serotype 6A/6B being the most common serotype among cultured strains [11]. However, there are no other reports from other regions in Indonesia describing the pneumococcal serotype and antimicrobial profile isolated from nasopharynx of patients with ARTI. Meanwhile, in Indonesia, 13-valent pneumococcal conjugate vaccine (PCV13) vaccine, containing 13 invasive serotypes as follows: 1, 3, 4, 5, 6A, 6B, 7F, 9V, 14, 19A, 19F, 18C, 23F, was introduced into routine infant immunization nationwide in 2022. Therefore, this study is important to support baseline community survey data in Indonesia before vaccine implementation. In this study, we have investigated the carriage rates, distribution of serotype, and antimicrobial profile of * S. pneumoniae* from children and adult patients in two primary health centres and a tertiary referral hospital in Manado, North Sulawesi, Indonesia between 2019 to 2020 before PCV13 vaccine introduction.

## Methods

### Nasopharyngeal swab specimen

We collected nasopharyngeal (NP) swab specimens from Puskesmas Tuminting and Puskesmas Bahu (primary health centres) and R. D. Kandou Hospital, a tertiary referral hospital in Manado, North Sulawesi, Indonesia between May 2019 to March 2020. The patients enrolled in the study included outpatients admitted to Tuminting and Bahu primary health centre sites and inpatients admitted to R. D. Kandou Hospital (Table S1). We enrolled the patients with fever, ≥37.5 °C, or history of fever with one or more of the respiratory symptoms: cough, nasal obstruction, rhinorrhea, and sore throat [12]. Data on demography, risk factors, and clinical symptoms were collected on day of admission by trained primary health centre or hospital staff.

NP swabs from patients were collected using a swab (flexible nasopharyngeal flocked swab; Catalogue no. 516CS01 for infant, Catalogue no. 503CS01 for subjects >1-year-old). Swab specimens were placed into STGG (1.0 ml of skim-milk, tryptone, glucose, and glycerol) transport medium and were shipped on ice pack box to the R. D. Kandou Hospital laboratory and were stored at −80 °C prior to testing.

### Bacterial isolation and identification

*Streptococcus pneumoniae* strains were isolated and identified as described previously [6]. Briefly, NP-STGG sample (volume=200 µl) was inoculated to 5 ml Todd-Hewitt-0.5 % yeast extract broth supplemented with 1 ml of rabbit serum followed by vortexing. The mixture was then incubated for 5 h at 37 °C with 5 % CO_2_. Thereafter, 10 µl of each mixture was plated onto an 8 % sheep blood agar plate with TSA II [BD] as base agar followed by incubation at 37 °C with 5 % CO_2_ for 18–20 h. The alpha-hemolytic colonies were subcultured and tested for optochin susceptibility. Gram-staining, and bile solubility tests were conducted to confirm the identification of *S. pneumoniae*. Gram-stain-positive, and optochin susceptible isolates were harvested into STGG and stored at −80 °C for further identification. DNA extraction was performed by enzymatic fast DNA extraction as as described previously [13]. Serotype determination was performed by a sequential multiplex PCR and Quellung reaction [14]. The isolate with bile solubility positive results but negative antisera reaction was classified as a non-typeable isolate.

### Antimicrobial profile testing

All *S. pneumoniae* isolates were tested for the antimicrobial profile using the disc diffusion method. The antimicrobial profile test was performed according to Clinical and Laboratory Standards Institute and on Mueller-Hinton agar with 5 % sheep blood using suspension prepared in Mueller-Hinton broth equal to 0.5 Mc Farland [6]. The antimicrobial test was included erythromycin [oxoid], chloramphenicol [oxoid], trimethoprim-sulfamethoxazole [oxoid], clindamycin [oxoid], azithromycin [oxoid], tetracycline [oxoid], and vancomycin [oxoid], Susceptibility to penicillin was tested with the oxacillin disc. *S. pneumoniae* ATCC 49619 was used as the control strain.

### Data analysis

The chi-square test was performed to compare the differences in the proportion of positive *S. pneumoniae* cultures among age, sex, symptoms, onset of fever, family member, chest X-ray, and diagnosis groups. SPSS Statistics 25 (IBM Corp, NY, USA) and Prism v.9.0.2 (GraphPad Software, La Jolla, CA, USA) were used to performed statistical analysis.

## Results

**Fig. 1.**
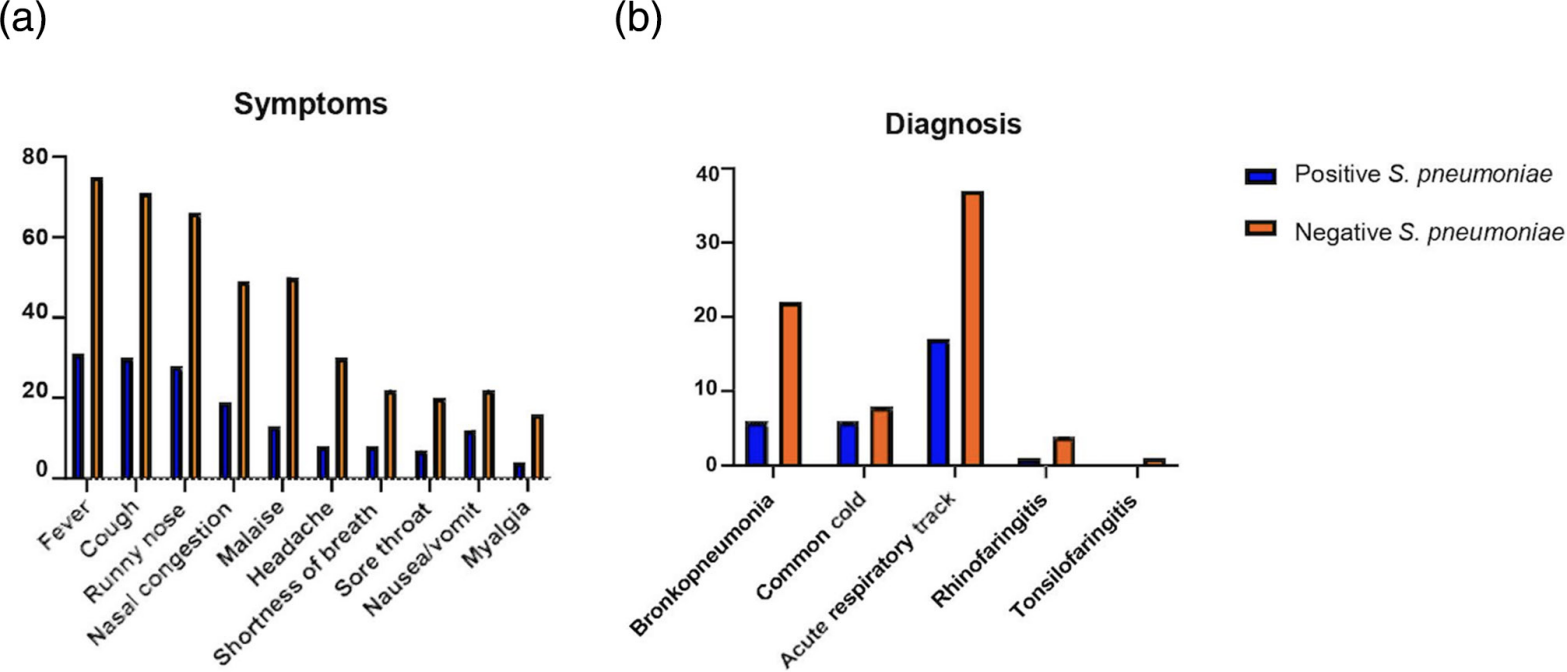
Graph (**a**) symptoms and (**b**) diagnosis of of patients with acute respiratory tract infections (number) in Manado, North Sulawesi, Indonesia.

**Fig. 2.**
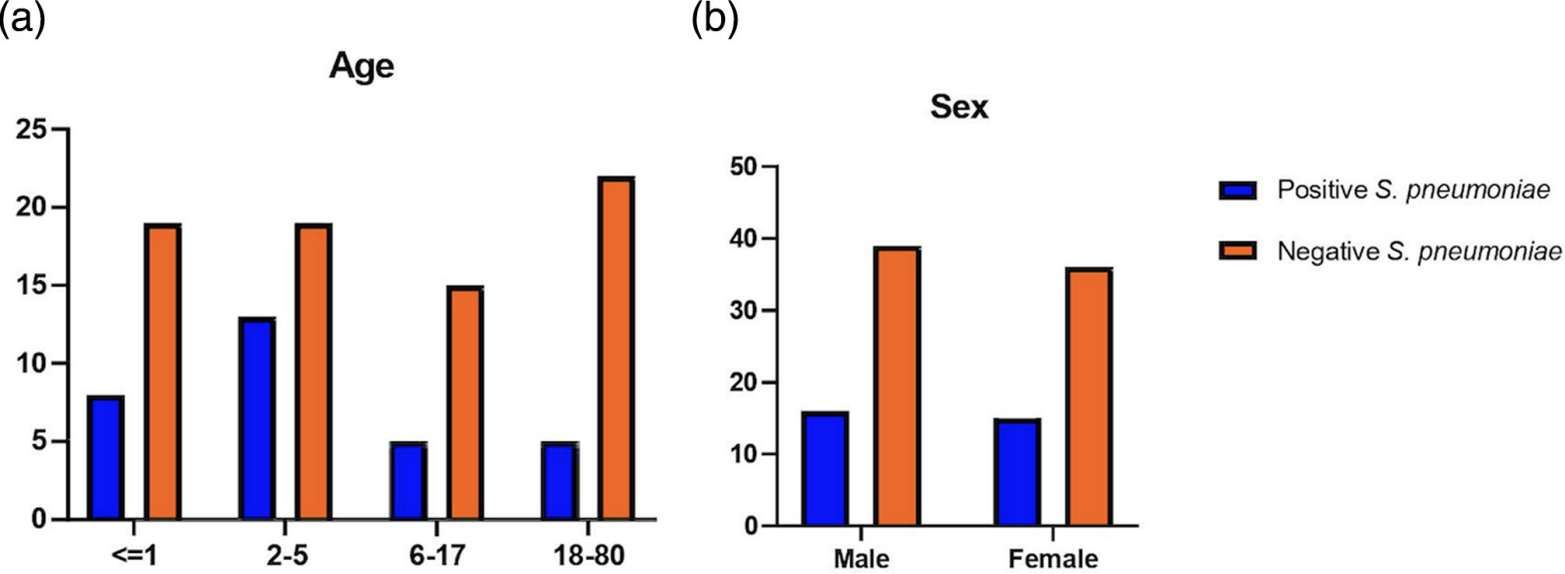
Graph (**a**) age and (**b**) sex of of patients with acute respiratory tract infections (number) in Manado, North Sulawesi, Indonesia.

We collected NP Swab specimens from 106 of patients with ARTI in Manado, North Sulawesi (Data S1). The patient characteristics are presented in (Table 1). In this study, the majority of the subjects were children and adolescents under 18 years of age (79/106, 74.5 %). We found that the common clinical symptoms were fever, cough, and runny nose (Fig. 1a). Chest X-ray data were available from 21 out of 30 patients from hospital, which were reported as infiltrates. Majority of patients were diagnosed with acute respiratory tract infection (50.9 %) followed by bronchopneumonia (26.4 %) and common cold (13.2 %) (Fig. 1b). We found that 31 patients carried *S. pneumoniae* strains (29 %). The nasopharyngeal carriage of *S. pneumoniae* was found to be higher among children aged 2–5 years (13/32; 40.6 %) than in children under 1 year (8/27; 29.6 %), children and adolescents under 18 years of age (5/20; 25.0 %) and adult patients (5/27; 18.5 %) (Fig. 2a). This study shows that number of men and women infected with pneumococcus was almost equal (Fig. 2b).

**Table 1.** Characteritics of patients with acute respiratory tract infections in Manado, North Sulawesi, Indonesia

Variables		Total N (%)	Total positive pneumococcus N (%)	*P* value
Age (years)				0.2981
	0–1	27 (25.5)	8 (29.6)	
	2–5	32 (30.2)	13 (40.6)	
	6–17	20 (18.9)	5 (25.0)	
	18–80	27 (25.5)	5 (18.5)	
Sex				
	Male	55 (51.9)	16 (29.1)	0.9711
	Female	51 (48.1)	15 (29.4)	
Type of facilities				
	Primary Health Centre (PHC)	74 (69.8)	25 (32.9)	0.1638
	Hospital	30 (28.3)	6 (20.0)	
Onset of fever (days)				0.8797
	1–5	99 (93.4)	29 (29.3)	
	>5	3 (2.8)	1 (33.3)	
Antibiotic use				
	Yes	12 (11.3)	2 (16.7)	N/A
Fuel type				
	Gas	94 (88.7)	29 (30.9)	0.5396
	Kerosene	7 (6.6)	1 (14.3)	
	Wood	2 (1.9)	1 (50.0)	
Smoking				
	Yes	7 (6.6)	2 (28.6)	N/A
Exposure to cigarete				N/A
	Yes	68 (64.2)	23 (33.8)	
Number of family members				
	1–3	17 (16.0)	4 (23.5)	0.7313
	4–6	67 (63.2	22 (32.8)	
	>6	18 (17.0)	5 (27.8)	
Symptoms				
	Fever	106 (100.0)	31 (29.2)	0.8691
	Cough	101 (95.3)	30 (28.3)	
	Runny nose	94 (88.7)	28 (26.4)	
	Nasal congestion	68 (64.2)	19 (17.9)	
	Malaise	63 (59.4)	13 (12.3)	
	Headache	38 (35.8)	8 (7.5)	
	Shortness of breath	30 (28.3)	8 (7.5)	
	Sore throat	27 (25.5)	7 (6.6)	
	Nausea/Vomit	34 (32.1)	12 (11.3)	
	Myalgia	20 (18.9)	4 (3.8)	
Chest X-ray result				
	Infiltrates	21 (19.8)	3 (2.8)	N/A
Diagnosis				
	Bronchopneumonia	28 (26.4)	6 (21.4)	0.5884
	Common cold	14 (13.2)	6 (42.9)	
	Acute respiratory infection	54 (50.9)	17 (31.5)	
	Rhinofaringitis	5 (4.7)	1 (20.0)	
	Tonsilofaringitis	1 (0.9)	0 (0.0)	

N = Total sample; *p*<0.05; N/A = Not Applicable.

In this study, we identified 31 *S*. *pneumoniae* strains. The distribution of serotypes varied, including 14, 18C, 19A, 23F, and 35B (two strains each) and 1, 3, 6B, 6C, 31, 9V, 15C, 16F, 17F, 23A, 19F and 35F (one strain each). Whereas, 29 % (9/31) of the isolates were non-typeable (Table 2). We found that vaccine type (13-valent pneumococcal conjugate vaccine, PCV13) strains were found higher in children under 5 years old (9/21; 43 %) than in children above 5 years old (4/13; 40 %) and adults (1/5; 20 %).

**Table 2.** Serotype distribution and antimicrobial profile of *Streptococcus pneumoniae* strains isolated from patients with acute respiratory tract infections in Manado, North Sulawesi, Indonesia

Isolate ID	Serotype	CHL	CLI	PEN*	AZM	SXT	ERY	VAN	TET
RMD.024	14	S	S	R	S	R	S	S	R
RMD.015	14	S	S	R	S	R	S	S	R
RMD.001	18C	S	S	R	R	R	R	S	S
RMD.028	18C	S	S	S	S	R	S	S	R
RMD.026	19A	S	S	S	S	S	S	S	S
RMD.208	19A	S	S	R	S	R	S	S	R
RMD.031	23F	S	S	R	S	S	R	S	R
RMD.221	23F	S	S	R	R	R	R	R	R
RMD.131	1	R	S	S	S	R	S	S	R
RMD.139	3	S	S	S	S	S	S	S	S
RMD.013	6B	S	S	R	S	R	S	S	S
RMD.222	19F	S	S	R	R	R	R	S	R
RMD.122	9V	S	S	R	R	R	R	S	R
RMD.027	35B	S	S	R	R	R	R	S	R
RMD.225	35B	S	R	R	R	R	R	S	R
RMD.020	6C	S	S	S	S	S	S	S	S
RMD.006	15C	S	S	R	S	S	S	S	S
RMD.009	16F	S	S	S	S	S	S	S	S
RMD.130	17F	S	R	S	R	R	S	S	R
RMD.035	23A	S	S	S	S	S	S	S	S
RMD.002	31	R	S	S	S	S	S	S	R
RMD.132	35F	S	S	S	S	S	S	S	S
RMD.003	Untypeable	S	S	R	S	R	S	S	R
RMD.014	Untypeable	S	S	R	S	R	S	S	S
RMD.107	Untypeable	S	S	R	R	R	R	S	R
RMD.112	Untypeable	S	S	R	R	R	R	S	R
RMD.005	Untypeable	S	S	R	S	R	S	S	S
RMD.033	Untypeable	S	S	S	S	S	S	S	S
RMD.037	Untypeable	S	S	R	S	R	S	S	R
RMD.216	Untypeable	S	S	R	S	R	S	S	S
RMD.232	Untypeable	S	S	R	S	R	S	S	S

Grey colour indicates: Vaccine serotypes (PCV13=1, 3, 4, 5, 6A, 6B, 7F, 9V, 14, 19A, 19F, 18C, 23F).

*Susceptibility to penicillin was tested with the oxacillin disk.

AZM Azythromycin CHL Chloramphenicol CLI Clindamycin ERY Erythromycin PEN Oxacillin SXT Sulphametoxazole/trimetrophim TET Tetracycline VAN Vancomycin

We found that the majority of the strains were susceptible to vancomycin (30/31; 97 %), chloramphenicol (29/31; 94 %), clindamycin (29/31; 94 %), erythromycin (22/31; 71 %), and azithromycin (22/31; 71 %). Meanwhile, the strains were less susceptible to tetracycline (14/31; 45 %), penicillin (11/31; 35 %), and sulfamethoxazole/trimethoprim (10/31; 32 %) (Table 2). Sixteen of the 31 isolates (52 %) expressed less susceptibility to three or more antimicrobial agents of different classes.

## Discussion

We identified that the *S. pneumoniae* carriage rate was 40.6 % among children under 5 years-old diagnosed with ARTI symptoms. The prevalence of *S. pneumoniae* carriage rate in Manado was less than previously reported in children in Lombok (48 %) [15], in HIV-infected children in Jakarta (46 %) [16], and children in Semarang, Central Java (43 %) [17]. However, the carriage rate of *S. pneumoniae* among adult patients in Manado (18.5 %) was higher than previously reported for adults 45–75 years of age in Semarang (11 %), adults in Jakarta (3 %) and HIV-infected adults in Jakarta (10 %) [5,17,19]. Furthermore, we observed that serotypes included in the PCV13 were the commonest serotypes found in this study (42 %) before PCV13 vaccine introduction in Indonesia, which was also in agreement with a previous study [5].

Among 42 % of vaccine serotypes found in this study, we identified serotype three as one of prevalent serotypes which was previously reported as a common serotype found among adult patients with community-acquired pneumonia in Jakarta, Indonesia [20]. Vaccine serotypes including serotype 3, 7F, 19A, 1, and 14 were reported as the most frequent serotypes found in adult patients with invasive pneumococcal disease in Portugal before PCV13 [21]. Serotype 14 of *S. pneumoniae* was the most prevalent aetiology of pneumococcal community-acquired pneumonia in children [22]. We detected that 42 % of serotypes in this study were covered by the PCV13. Serotype analysis of pneumococcal nasopharyngeal carriage isolates in Indonesia revealed that 38–60 % of isolates are covered by the PCV13 [5].

There was an increasing number of non-susceptible *S. pneumoniae* strains to sulfamethoxazole/trimethoprim from 38 % in 2010 [15] to 68 % indicated in this study. Sulfamethoxazole/trimethoprim is the second most common antimicrobial prescribed for children and is included in the antibacterials that have to be reconsidered by physicians for use in public healthcare facilities in Indonesia [23]. Penicillin and sulfonamides are the most common classes of antibiotics consumed by infants from birth to 18 months of age (38.81 and 24.48 %, respectively) [24], while penicillin and tetracyclines accounted for 80 % of the prescribed antibiotics in public healthcare facilities [23].

In addition, a variety of factors associated with pneumococcal carriage were analysed in this study for their carriage rates among patients. Factors such as younger age, greater family size, and exposure to cigarette smoke have been reported positively associated with pneumococcal carriage while factors such as antibiotics usage have otherwise been negatively associated [25].

Carriage prevalence of *S. pneumoniae* varies among age groups. Children under the age of five had a greater pneumococcal nasopharyngeal carriage rate compared to other age groups classified in this study. In this study, the carriage rate for children under the age of five was 37 %, followed by 24 % for children in the age range of 12–17 and 18 % for adults 18 years old and above. Children are repeatedly reported as having higher carriage rates compared to adults, and the highest rates reported are often among children of 2 years of age [4,25]. A carriage study previously conducted in Kotabaru, South Kalimantan, Indonesia had relatively high carriage rates among children under 5 years old, ranging from 42.7–51.4 % [6]. Another survey conducted in Nigeria also reported their highest carriage rates among children ages 0–4, 33.9 % in Kumbotso and 36.5 % in Pakoto [26].

Patients having more family members also had a higher pneumococcal nasopharyngeal carriage rate compared to patients having fewer. In this study, patients having family members of 4–6 had a carriage rate of 32 %. The same carriage rate was also observed in patients having seven family members and above. However, patients having 1–3 family members had a lower carriage rate of 24 %. Increase in pneumococcal colonization in patients having larger family size have previously been observed as well in children who have siblings [27,28].

Furthermore, patients exposed to cigarette smoke had a higher risk of pneumococcal colonization in their nasopharynx. Cigarette smoke has previously been reported to increase susceptibility to colonization by bacterial pathogens such as * S. pneumoniae* and *H. influenzae* in the upper airways [29]. In this study, the carriage rate of paediatric patients exposed to cigarette smoke was 35 % while those not exposed to cigarette smoke had a carriage rate of 31 %.

Meanwhile, antibiotics usage has been observed to reduce the odds of pneumococcal colonization. Antibiotics use have been reported in a review as being protective against pneumococcal carriage [25]. In this study, patients that were on antibiotics during the period of study, had a carriage rate of 17 % while patients that were not using antibiotics had a carriage rate of 31 %. Another pneumococcal carriage study conducted in Kilifi district, Kenya have also observed lower odds of carriage in children who reported taking antibiotics in the previous 2 weeks of study [30].

This study described serotype distribution and antimicrobial profile among adult patients with ARTI only with nasopharyngeal specimens. The combination of other invasive clinical specimens representing the ARTI cases would elaborate better understanding on correlation of colonization and ARTI. We declared this as limitation of the study.

This study provides supporting baseline data on distribution of serotype and antimicrobial profile of *S. pneumoniae* among acute respiratory infections patients before PCV13 vaccine introduction in Manado, North Sulawesi, Indonesia.

## supplementary material

10.1099/acmi.0.000703.v4 Table S1.

## Abbreviations

ARTI: acute respiratory tract infections
NP: nasopharyngeal
PCV13: 13-valent pneumococcal conjugate vaccine
STGG: skim-milk, tryptone, glucose, and glycerol
